# Supplementary material for: Expression of PD-1, PD-L1 and PD-L2 in Lymphomas in Patients with Pre-Existing Rheumatic Diseases—A Possible Association with High Rheumatoid Arthritis Disease Activity
Source: Cancers (Basel). 2022 Mar 15;14(6):1509. doi: 10.3390/cancers14061509 (PMC8946311; doi:10.3390/cancers14061509)
Supplement: Supplementary file 1 [file cancers-14-01509-s001.zip › cancers-1598911-supplementary.pdf]

Supplementary Materials:

Expression of PD-1, PD-L1 and PD-L2 in Lymphomas in Patients with Pre-Existing Rheumatic Diseases—A Possible Association with High Rheumatoid Arthritis Disease Activity

Erik Hellbacher [1,*], Christer Sundström [2], Daniel Molin [3], Eva Baecklund [1] and Peter Hollander [2]

Table S1. DMARDs used in all RA-DLBCL patients at lymphoma diagnosis[1] and comparison between the highest RA disease activity group and the lower disease activity group.

	Entire cohort	RA highest disease activity group	RA lower disease activity group	P-value *
All patients	99 (100)	25 (100)	74 (100)	
Prorecid (podophyllotoxin)	6 (6)	0 (0)	6 (8)	0.33
Antimalarials	4 (4)	3 (12)	1 (1.5)	0.049
Sulfasalazine	4 (4)	1 (4)	3 (4)	1
Methotrexate	3 (3)	1 (4)	2 (3)	1
Chlorambucil	2 (2)	0 (0)	2 (3)	1
Azathioprine	2 (2)	1 (4)	1 (1.5)	0.44
Auranofin	1 (1)	0 (0)	1 (1.5)	1
Gold injection	1 (1)	0 (0)	1 (1.5)	1
Penicillamine	1 (1)	0 (0)	1 (1.5)	1

DMARD, disease modifying anti-rheumatic drugs; RA, rheumatoid arthritis; DLBCL, diffuse large B-cell lymphoma. [1]Defined as the treatment at the time of lymphoma diagnosis provided it had been used for four or more consecutive weeks. * According to Fischer's exact test.

Table S2. Comparison of median proportions of PD-L1+ tumor cells and TILs among the various lymphoma subtypes.

Lymphoma subtype	Number of cases *	Median proportion of PD-L1+ tumor cells, (range)	Median proportion of PD-L1+ TILs, (range)
cHL	13	16.0 (0-75.7)	19.6 (0.3-63.1)
DLBCL	112	1.6 (0-85.1)	10.2 (0-89.3)
FL	32	0.0 (0-6.1)	7.4 (0.2-38.5)
CLL	13	0.0 (0-7.9)	1.4 (0.3-9.8)
LPL	3	0.6 (0-46.2)	3.9 (0.7-53.2)
MZBL	5	0.7 (0-6.5)	3.2 (0.4-17.5)
MCL	5	0.0 (0-1.1)	4.3 (0.2-6.7)
Burkitt lymphoma	3	0.0 (0-0.2)	2.8 (2.0-3.8)
HGBCL	8	4.3 (0-30.4)	5.6 (0.5-21.5)
LGBCL	6	0.2 (0-13.0)	4.4 (1.5-.2.7)
TCL	5	4.2 (0-61.6)	2.8 (0-7.3)

* Number of cases with information enabling evaluation of PD-L1; cHL, classical Hodgkin lymphoma; DLBCL, diffuse large B-cell lymphoma; FL, follicular lymphoma; CLL, chronic lymphocytic leukemia; LPL, lymphoplasmacytic lymphoma; MZBL, marginal zone B-cell lymphoma; MCL, mantle cell lymphoma; HGBCL, high-grade B-cell lymphoma; LGBCL, low-grade B-cell lymphoma; TCL, T-cell lymphoma.

Table S3. Comparison of median proportions of PD-L2+ tumor cells and TILs among the various lymphoma subtypes.

Lymphoma subtype	Number of cases *	Median proportion of PD-L2+ tumor cells, % (range)	Median proportion of PD-L2+ TILs, % (range)
cHL	13	0.0 (0-51.5)	0.3 (0-14.9)
DLBCL	112	0.0 (0-32.3)	0.6 (0-56.5)
FL	32	0.0 (0-42.7)	12.1 (0.2-49.4)
CLL	13	0.0 (0-0.4)	0.5 (0-3.2)
LPL	3	1.2 (0.2-16.8)	18.5 (2.6-34.0)
MZBL	5	1.9 (0-3.8)	5.2 (0.4-15.6)
MCL	5	0.1 (0-5.7)	0.8 (0.3-5.6)
Burkitt lymphoma	3	0.0 (0)	0.1 (0-0.2)
HGBCL	8	0.1 (0-1.0)	0.6 (0.2-7.1)
LGBCL	6	0.0 (0-0.8)	0.8 (0.2-12.1)
TCL	5	0.2 (0-10.5)	1.8 (0-30.3)

* Number of cases with information enabling evaluation of PD-L2; cHL, classical Hodgkin lymphoma; DLBCL, diffuse large B-cell lymphoma; FL, follicular lymphoma; CLL, chronic lymphocytic leukemia; LPL, lymphoplasmacytic lymphoma; MZBL, marginal zone B-cell lymphoma; MCL, mantle cell lymphoma; HGBCL, high-grade B-cell lymphoma; LGBCL, low-grade B-cell lymphoma; TCL, T-cell lymphoma.

Table S4. Comparison of median proportions of PD-1+ TILs among the various lymphoma subtypes.

Lymphoma subtype	Number of cases *	Median number of PD-1+ TILs per HPF (range)
cHL	15	53.1 (0-296.9)
DLBCL	111	42.4 (0.4-560.9)
FL	29	236.3 (3.4-714.0)
CLL	13	56.6 (1.6-166.0)
LPL	4	36.9 (13.7-124.2)
MZBL	5	187.9 (183.8-611.6)
MCL	4	30.6 (8.8-80.6)
Burkitt lymphoma	3	18.5 (1.5-123.3)
HGBCL	8	28.6 (7.6-94.1)
LGBCL	6	91.6 (3.2-513.6)
TCL	5	9.9 (0-410.9)

* Number of cases with information enabling evaluation of PD-1; cHL, classical Hodgkin lymphoma; DLBCL, diffuse large B-cell lymphoma; FL, follicular lymphoma; CLL, chronic lymphocytic leukemia; LPL, lymphoplasmacytic lymphoma. MZBL, marginal zone B-cell lymphoma; MCL, mantle cell lymphoma; HGBCL, high-grade B-cell lymphoma; LGBCL, low-grade B-cell lymphoma; TCL, T-cell lymphoma.
